# Structure and Phase Composition of the Products Derived from Vacuum–Thermal Treatment of a Tellurium-Containing Middling

**DOI:** 10.3390/ma18194620

**Published:** 2025-10-06

**Authors:** Alina Nitsenko, Xeniya Linnik, Valeriy Volodin, Sergey Trebukhov, Bulat Sukurov, Farkhad Tuleutay, Tolebi Dzhienalyev

**Affiliations:** Institute of Metallurgy and Ore Beneficiation JSC, Satbayev University, Almaty 050010, Kazakhstan; alina.nitsenko@gmail.com (A.N.); volodinv_n@mail.ru (V.V.); s.trebukhov@satbayev.university (S.T.); bsukurov@gmail.com (B.S.); farkhat_kaldybek@mail.ru (F.T.);

**Keywords:** copper telluride, vacuum, condensate, phase composition

## Abstract

In this paper, the results from a study of the products obtained by vacuum–thermal processing of industrial copper telluride in an inert atmosphere at a pressure of 66 Pa and a temperature of 1100 °C are presented. The residue obtained mainly consisted of the copper(I) oxide phase. The condensate was represented by the phases CuTe_2_O_5_, CuO·CuTeO_3_, TeO_2_, SiO_2_, and CuTe_2_Cl. The vapor phase condensed in four temperature zones, each represented by a different phase composition. A monophase of tellurium oxide was identified in the condensate at temperatures of 150 to 270 °C. The obtained data contribute to expanding scientific knowledge and form the basis for developing a new, environmentally safe method of processing tellurium-containing middling. The creation of new technologies promotes increased efficiency of tellurium recovery and reduces environmental risks.

## 1. Introduction

The main raw material used as a source of tellurium is the slime from copper electrolysis production. A fairly wide range of technologies has been proposed for its processing [1,2,3,4,5,6,7], accounting for the complexity and diversity of the chemical and phase compositions of the slimes. In the traditional approach [8,9,10], the first stage of slime processing is leaching, as a result of which tellurium is concentrated in solution. Selenium and silver are first extracted from the solution. Then, tellurium is precipitated as a finished middling product, copper telluride, by adding copper to the solution. Industrial copper telluride is a mixture of phases of both stoichiometric (Cu_2_Te) and non-stoichiometric compositions (Cu_2−x_Te) [11,12,13,14,15], and it also contains a small amount of impurities (other elements and compounds).

A classical method of processing tellurium-containing middling is oxidative-alkaline leaching with the addition of NaOH [9,10,16]. Using this method, a solution containing tellurium in the form of Na_2_TeO_3_ is obtained, which is directed to electrolysis along with a copper-containing residue. Recently, alternative hydrometallurgical methods aimed at improving upon the classical method to increase the leaching efficiency have been developed [14,17,18].

Pyrometallurgical methods have developed relatively recently. This is due to the fact that the decomposition of Cu_2_Te into copper and tellurium is only possible at temperatures above 2704 °C [8,19,20,21,22]. Decomposition of Cu_2_Te under real conditions by a vacuum–thermal method is also not feasible, due to the low dissociation pressure of liquid copper telluride: 700 Pa at 1780 °C. One option for developing pyrometallurgical methods for processing copper telluride is producing elemental tellurium through intermediate products.

It should be noted that the use of a vacuum improves working conditions for personnel and the environment. This is especially important when processing raw materials containing toxic elements such as tellurium. Tellurium compounds (hydrogen telluride, tellurous and telluric acids and solutions of their salts, sodium tellurite, and tellurium oxide) are highly toxic respiratory irritants that have the potential to cause chronic poisoning. Conducting the process in a sealed apparatus and capturing the tellurium-containing compounds through condensation significantly reduces the environmental risks associated with the toxicity of tellurium and its compounds.

Thus, in previous study of Li Zh. et al. [23], we proposed using directional sulfidation-vacuum distillation to extract tellurium from copper telluride. In this method, elemental sulfur in powder form was used as the sulfidizer, which was mixed with copper telluride. The obtained charge was briquetted. Sulfidation was performed by at atmospheric pressure in an inert atmosphere. Tellurium sublimation from the sulfide material was performed at a pressure of 10–20 Pa. Such a low pressure enabled extraction of up to 97% of the tellurium at a purity of 96.37% at a temperature of 650 °C. All copper in the residue from vacuuming was present in the sulfide form.

An oxidative-distillation method was also proposed by Nitsenko A. et al. [24], which involves converting copper telluride into copper orthotellurate, the decomposition of which produces tellurium oxide (TeO_2_). Tellurium oxide passes into the vapor phase and condenses at low temperature into a solid crystalline phase due to its high vapor pressure at the roasting temperature. A technologically acceptable degree of tellurium recovery in the condensate (98%) was achieved at 1100 °C (pressure 670 Pa, oxidant flow rate (air) 2.2 × 10^−2^ m^3^/m^2^·s, roasting duration 60 min). The main phase of the condensate is tellurium oxide, which can be processed in a single step into elemental chalcogen by thermal reduction or electrolysis.

In work of Nitsenko A. et al. [25], it was shown that the curves of mass loss of industrial tellurium-containing middling versus temperature in oxidative and inert atmospheres have a similar character. In addition, the final degree of mass loss had the same value (about 21%). Oxygen was found in amounts from 18% (at delivery) to 35% (after storage for two years) when studying the composition of technical copper telluride [13,15,23,24]. Therefore, the most reasonable explanation for mass loss in an inert atmosphere, apart from dehydration, is the presence of oxidizers in the material itself.

Thus, we performed a series of studies aimed at determining whether tellurium could be extracted from tellurium-containing middling by a vacuum–thermal method in an inert atmosphere without the use of additional reagents.

In an earlier study of Nitsenko A. et al. [26], we reported the thermal behavior of tellurium-containing middling under vacuum–thermal treatment in an inert atmosphere. By means of X-ray phase analysis, it was shown that the formation of tellurium oxide occurs due to oxidation processes involving the oxygen contained in the material. Through electron probe microanalyses (EPMA), it was determined that industrial copper telluride was oxidized through intermediate compounds to Cu_3_TeO_6_ at low pressure, which decomposed through CuTeO_3_ to CuO and TeO_2_ at 900 °C. The results of technological tests showed that lowering the pressure and/or increasing the temperature had a positive effect on the degree of tellurium recovery in the condensate. A tellurium recovery rate of 98.70% was achieved at a temperature of 1100 °C and a pressure of 66 Pa.

The present study is a continuation of [26], and is aimed at studying the phase composition and structure of the products obtained by vacuum–thermal processing of tellurium-containing middling at 1100 °C and 66 Pa. The phase and elemental compositions of the residues obtained at various temperatures and at a pressure of 66 Pa under isothermal conditions were also analyzed. The information presented in this paper contributes to expanding scientific knowledge about the behavior of technical copper telluride during vacuum–thermal processing in an inert atmosphere and provides complete characterization of the resulting products.

## 2. Materials and Methods

### 2.1. Materials

This study used a tellurium-containing middling from Kazakhmys Smelting LLP (Balkhash, the Republic of Kazakhstan), oxidized under natural conditions (storage period is 1 year).

**Figure 1:**
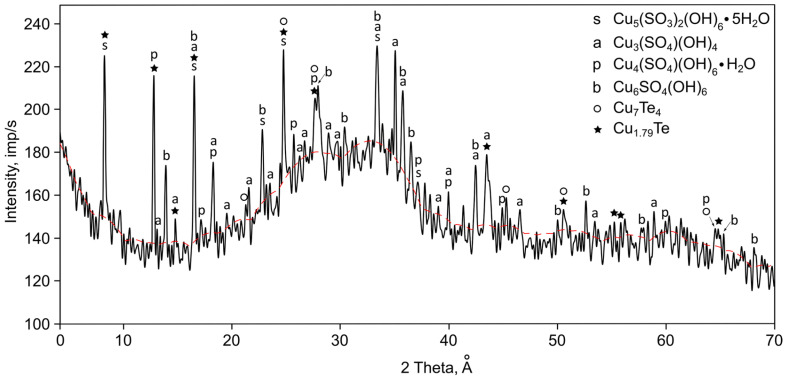
X-ray diffractogram of the tellurium-containing middling from Kazakhmys Corp., LLP.

Copper telluride is an odorless agglomerated material that is malachite in color. The moisture content was 3%. The material composition is presented in Table 1. According to the results from the X-ray diffraction analysis (Figure 1), a large proportion of an amorphous halo caused by scattering from disordered phases was identified. The crystalline part is represented by phases of non-stoichiometric copper tellurides (Cu_7_Te_4_—PDF 00-057-0196, Cu _1.79_Te—PDF 01-082-9896) and copper hydroxysulfates (Cu_5_(SO_3_)_2_(OH)_6_·5H_2_O—PDF 00-041-0007, Cu_3_(SO_4_)(OH)_4_—PDF 00-007-0407, Cu_4_(SO_4_)(OH)_6_·H_2_O—PDF 01-083-1410, Cu_6_SO_4_(OH)_6_—PDF 00-043-1458). The presence of the latter can be explained by insufficient washing of copper telluride from the CuSO_4_ solution after tellurium cementation on copper from tellurous acid.

### 2.2. Methodology

**Figure 2:**
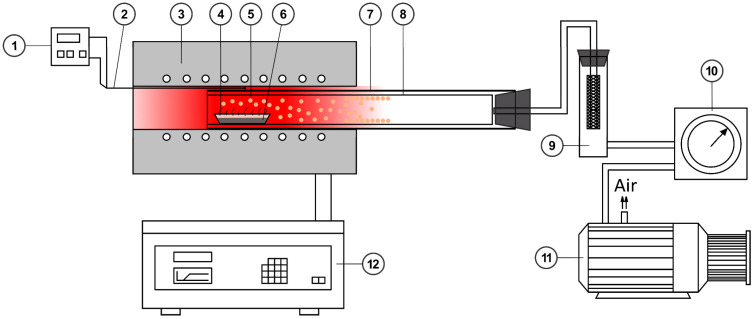
Horizontal vacuum setup with a detachable condenser: (1) temperature controller in the reaction zone, (2) control thermocouple, (3) electric furnace, (4) boat, (5) isothermal zone, (6) sample, (7) reactor, (8) detachable condenser, (9) filter, (10) barometer and manometer, (11) vacuum pump, (12) furnace controller.

A laboratory setup with a horizontally arranged reactor was used for this study (Figure 2). The setup consisted of a tubular electric furnace RT 50/250/13 (Nabertherm Lilienthal, Germany) with a B-180 controller, in which the reactor and gas evacuation system were placed. A vacuum pump 2NVR-5DM UKhL4 (Vakuummash, Kazan, Russia) was used to create reduced pressure in the system. The pressure was measured with a DCP 3000 vacuum gauge (Vacuubrand, Wertheim, Germany) and a VSP 3000 sensor (accuracy ±10 Pa). A filter was installed at the outlet of the retort to capture particles not deposited in the reactor. An additional chromel–alumel thermocouple (thermoelectric transducer DTPK021-1.2/0.7) with a single-channel microprocessor-based measuring controller TRM1 was used to control the temperature in the reaction zone.

The reactor is a quartz vessel containing an alumina boat with a weighed portion of the sample. A detachable quartz condenser was placed over the boat to collect the condensed material. A weighed portion of copper telluride of the required mass was loaded into the boat during the experiment. The boat was then placed into the detachable (longitudinal) condenser. The condenser was in turn placed into the quartz reactor. The reactor was connected to the vacuum system and then purged several times with an inert gas (argon). After evacuating the gases from the system to the specified pressure, the reactor was placed into the preheated furnace in such a way that the copper telluride sample was located in the isothermal zone. At the end of the experiment, the reactor was removed from the furnace and cooled under vacuum. The obtained products were weighed and analyzed. Weighing was performed using PA214C analytical balances (Ohaus-Pioneer, Parsippany, NJ, USA) with an accuracy of ±0.1 mg.

### 2.3. Characterizations

The material composition was studied via X-ray fluorescence analysis using a wavelength-dispersive spectrometer Axios 1 kW (PANalytical, Almelo, The Netherlands) with an accuracy of ±5%.

For phase composition identification, X-ray diffraction analysis was performed using a D8 Advance diffractometer (Bruker, Bremen, Germany) and Cu-Kα radiation. The phase composition was determined using the ASTM database (reference database of diffraction data PDF-2 rel. 2023 of the International Centre for Diffraction Data (ICDD, Newtown Square, PA, USA)).

Electron probe microanalysis (EPMA) was performed using a JXA-8230 electron probe microanalyzer (JEOL, Tokyo, Japan) in energy-dispersive spectrometry (EDS) mode under the following conditions: accelerating voltage –20 kV; current—5 nA.

## 3. Results and Discussion

### 3.1. Assessment of Possible Phase Transformations

It is important to take into account the vapor pressure of elements and compounds prone to evaporation under the selected conditions when analyzing thermal processes in vacuum. The presence of tellurium, oxygen, copper, and chlorine in the studied material indicates the possibility of forming various phases during vacuum–thermal treatment, each of which will have characteristic ranges of stability and vapor pressure.

It is known [27] that the oxidation of synthetically obtained copper telluride by oxygen supplied externally into the reaction medium proceeds according to the reaction:

Cu_2_Te + 1/2O_2_ = 2Cu_2_O + Te, (1)

When a sufficient amount of tellurium accumulates, it undergoes oxidation to TeO_2_, which, before reaching the evaporation temperature, reacts with copper oxides to form CuTe_2_O_5_ and/or CuTeO_3_.

Under real conditions, partial oxidation of the tellurium-containing middling, due to technological process disturbances in production, is possible. Such oxidation is characterized by the presence of copper hydroxysulfates in the material. Therefore, the oxidation of copper telluride is possible due to the oxygen contained in the material and released during phase transformations.

Based on the phase and elemental compositions of the studied intermediate, it can be assumed that, at the initial stage of thermal treatment, molecular water will be removed from the crystalline structures of Cu_5_(SO_3_)_2_(OH)_6_·5H_2_O and Cu_4_(SO_4_)(OH)_6_·H_2_O, which will lead to an increase in the content of the brochantite and antlerite phases. Brochantite (Cu_4_(SO_4_)(OH)_6_), belonging to the class of basic copper(II) sulfates, in the temperature range of 250–400 °C [28], will decompose with the formation of antlerite (Cu_3_(SO_4_)(OH)_4_), copper(II) oxide, and water. In turn, antlerite will decompose to CuO, sulfur oxide (SO_2_), residual water, and oxygen at temperatures above 600 °C [29]. Dolerophanite (Cu_2_O(SO_4_)) [29,30] may also be one of the decomposition products of brochantite and antlerite. According to [31], dolerophanite decomposes at temperatures above 600 °C with the formation of CuO and SO_3_.

We have established that the copper(II) oxide formed according to this scheme, at moderate temperatures, is capable of reacting with chlorine contained in the material to form copper chloride (CuCl_2_) and oxygen [26]. The oxygen released during the above-mentioned phase transformations will oxidize copper telluride according to reaction (1) and then oxidize elemental tellurium to its oxide. Chlorine can react with elemental tellurium to form tetrachloride (TeCl_4_), in addition to interacting with copper(II) oxide.

**Figure 3:**
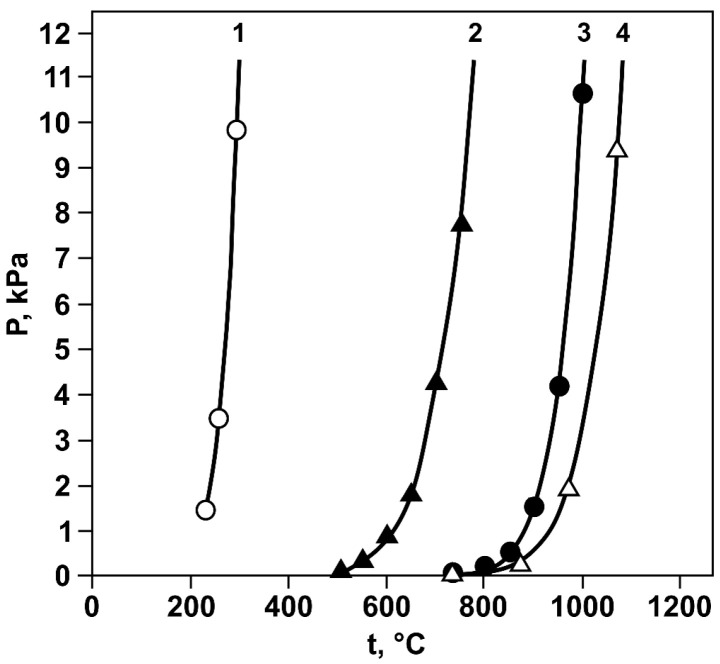
Dependence of the saturated vapor of TeCl_4_ (1), Te (2), and TeO_2_ (3, 4) [26].

Thus, the formation of volatile tellurium-containing compounds, as well as tellurium in elemental form, should be expected during vacuum–thermal treatment of tellurium-containing middling in an inert atmosphere. The assumption of the possibility of elemental tellurium evaporation is based on an insufficient amount of oxygen being present to complete the oxidation of tellurium to dioxide. Figure 3 shows the temperature dependencies of the vapor pressure of elemental tellurium and tellurium-containing compounds. The graph was constructed using data from [8].

As can be seen from the figure, tellurium tetrachloride is the most volatile tellurium-containing compound. Tellurium dioxide is weakly volatile up to its melting temperature. Therefore, it should be expected that TeO_2_ evaporation will occur after decomposition of the intermediate tellurate and tellurite phases formed during the interaction of copper and tellurium oxides in the temperature range of 300 to 650 °C.

### 3.2. Phase Transformations of Tellurium-Containing Middling

**Figure 4:**
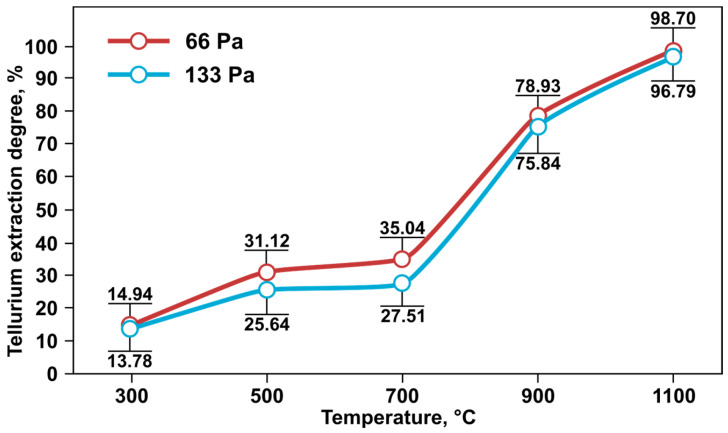
Dependence of the degree of tellurium evaporation on constant temperature at pressures of 66 and 133 Pa.

As shown in [26], a decrease in pressure and/or an increase in temperature predictably leads to an increase in the degree of tellurium recovery. The best results for tellurium evaporation were achieved at a temperature of 1100 °C: 98.70% at 66 Pa and 96.79% at 133 Pa. The tellurium content in the residues after vacuum treatment under these conditions amounted to 0.72% at 66 Pa and 1.62% at 133 Pa (Table 2). At the same time, a noticeable increase in the degree of tellurium evaporation was observed at temperatures above 700 °C, which is associated with reaching the evaporation temperature of tellurium oxide compounds (Figure 4).

**Figure 5:**
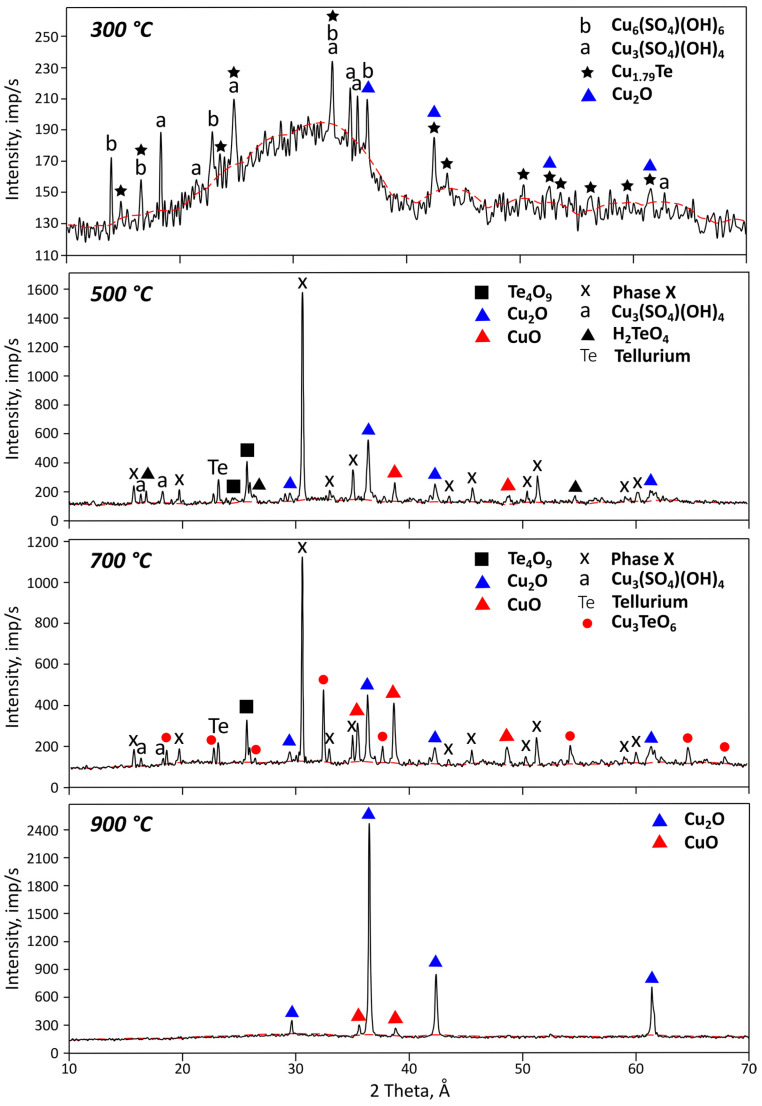
X-ray diffractograms of residues obtained at 66 Pa under isothermal conditions.

Figure 5 shows the X-ray diffractogram of the residues obtained under isothermal conditions at a pressure of 66 Pa.

X-ray phase analysis of the residues obtained at 300 °C also confirmed the decomposition of posnjakite (Cu_4_(SO_4_)(OH)_4_) and copper hydroxysulfate hydrate (Cu_5_(SO_3_)_2_(OH)_6_·5H_2_O) into brochantite (Cu_6_SO_4_(OH)_6_) and antlerite (Cu_3_(SO_4_)(OH)_4_). Complete decomposition of brochantite into antlerite occurred in the second temperature range (300–500 °C), while antlerite decomposed in the third (500–700 °C).

Due to the strong amorphous character of the material at 300 °C, in addition to antlerite and brochantite, it was possible to identify phases of telluride, copper, and copper oxide (Cu_2_O). The formation of the latter is apparently associated with the onset of copper telluride oxidation. The vacuum residue was sufficiently well crystallized at 500 °C. The X-ray diffractograms showed pronounced reflections of an unidentified phase X, presumably Cu_0.37_Te_0.26_O_0.76_S_0.084_, along with copper and tellurium oxides. Phase X was present in predominant amounts. Additional studies using a pure compound are required to determine the nature of this compound and the conditions of its formation and decomposition. At 700 °C, the formation of copper orthotellurate (Cu_3_TeO_6_), which is stable up to 880 °C, according to the literature [31,32], was observed, along with the presence of a small amount of elemental tellurium. At 900 °C, the residues were represented by copper oxide phases. Previously [26], it was shown that, according to electron probe microanalysis, the residues obtained at 900 °C concentrated tellurium in a compound not detected by X-ray phase analysis, with a composition close to CuTeO_3_.

### 3.3. Analysis of Vacuum–Thermal Treatment Products Formed at 1100 °C

#### 3.3.1. Residues

**Figure 6:**
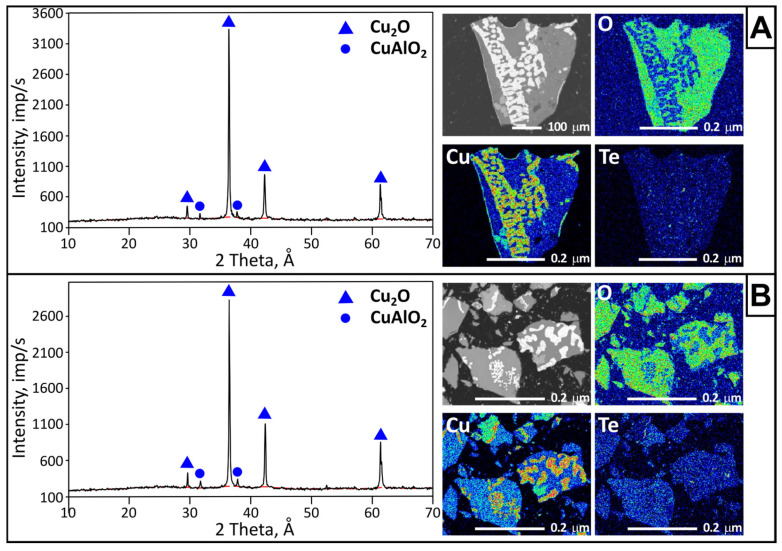
X-ray phase and electron probe microanalyses of residues obtained at 66 Pa (**A**) and 133 Pa (**B**).

Visually, the residues obtained at 1100 °C represent a fused material with traces of boiling. X-ray phase analysis of the crystalline phases (Figure 6) revealed the presence of copper(I) oxide and copper aluminate CuAlO_2_ (copper aluminum oxide). The content of copper(I) oxide varies from 75 to 85 wt.%.

Copper(I) oxide is a product of the thermal reduction of copper(II) oxide in an oxygen-free environment. The formation of copper aluminate can occur through the interaction of both CuO and Cu_2_O with the alumina crucible material, according to the reactions 2CuO + Al_2_O_3_ → 2 CuAlO_2_ + 1/2 O_2_ [33] and Cu_2_O + Al_2_O_3_ → 2 CuAlO_2_ [34]. During process scaling, it is possible to prevent the formation of aluminate by changing the crucible material or by forming a protective (garnishing) layer.

According to electron probe microanalysis (Figure 6), the alloy matrix (gray) represents a complex mixture consisting of oxygen (39.49 to 46.63 wt.%), aluminum (7.66 to 10.31 wt.%), silicon (16.25 to 21.31 wt.%), copper (19.20 to 30.13 wt.%), and tellurium (0.43 to 1.34 wt.%). Copper oxide is present as irregular inclusions (white), with copper content at measurement points ranging from 86.25 to 86.92 wt.%. Copper aluminate (Cu—49.50 to 52.50 wt.%; Al—18.57 to 19.15 wt.%; O—28.62 to 30.71 wt.%) occurs in the matrix as separate grains (light gray). EDS mapping also revealed the presence of silicon oxide (dark gray). Residual tellurium is evenly distributed throughout the mass of the alloy.

#### 3.3.2. Condensate

**Figure 7:**
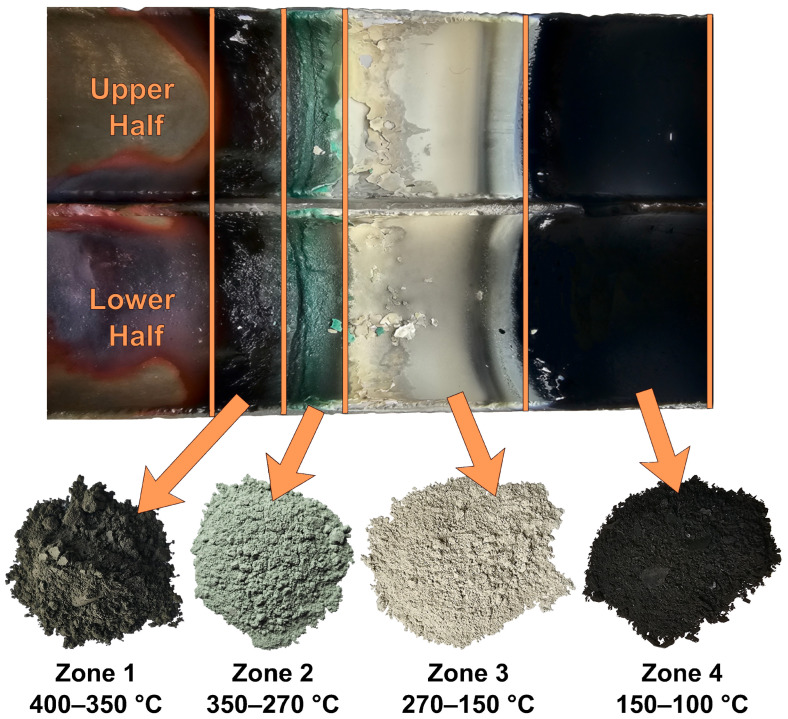
Appearance of the condensates.

Condensate deposition occurred in the “cold” part of the reactor within the temperature range of 100 to 400 °C. Several deposition zones with different colors were observed on the condenser (Figure 7) by the end of the process. Black condensate was deposited in the hot condensation zone (400–350 °C). As the temperature decreased, zones of dark green (350–270 °C) and beige (270–150 °C) were observed. Fine black condensate was deposited in the final condensation zone (150–100 °C).

According to the X-ray phase analysis results, the overall condensate sample consisted of the following phases: TeO_2_ (PDF 01-074-0269)—60%; Te_3_Cu_2_O_7_ (PDF 00-035-1083)—24%; and CuTe_2_O_5_ (PDF 00-033-0494)—16%. X-ray fluorescence analysis showed that the main elemental composition (wt.%) was Te—62.066; Cu—8.449; and O—24.887. Silicon (3.022 wt.%) was also present, introduced during sample collection from the condenser surface. Minor amounts (up to 0.4 wt.%) of aluminum, sulfur, chlorine, potassium, chromium, selenium, and arsenic were detected.

**Figure 8:**
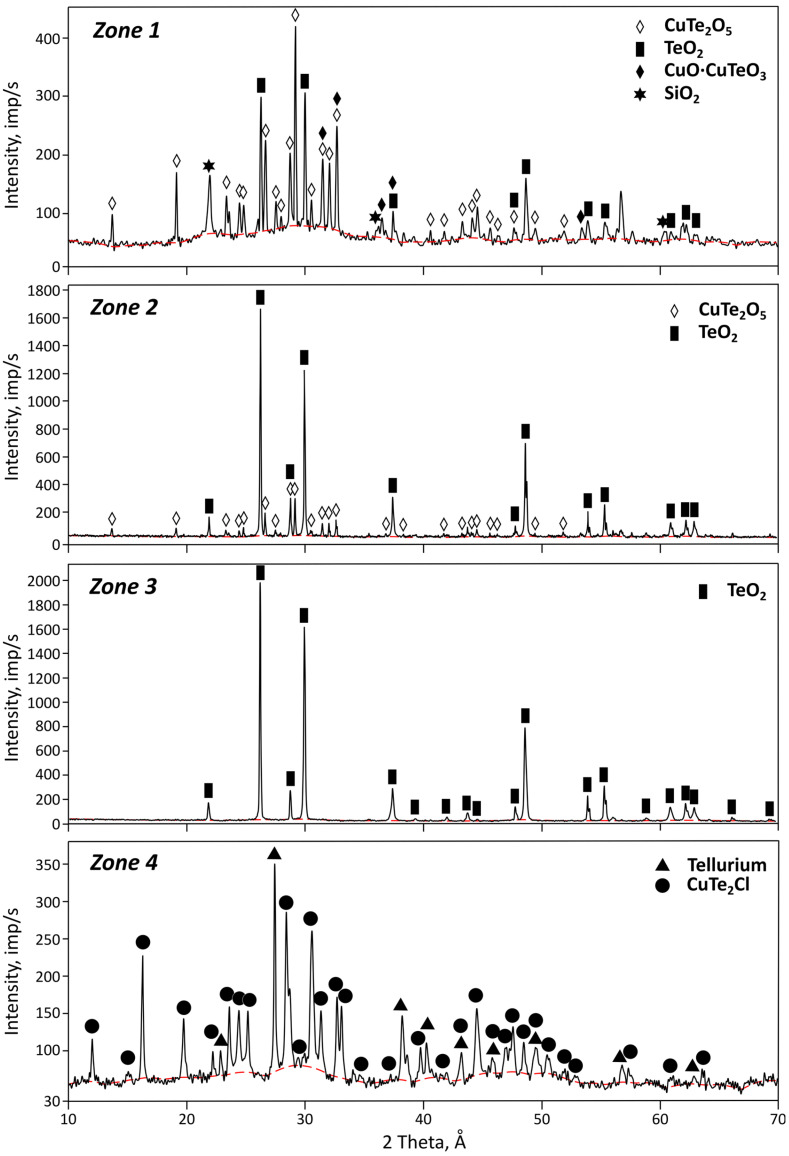
X-ray diffractograms of condensates collected from different temperature zones.

**Figure 9:**
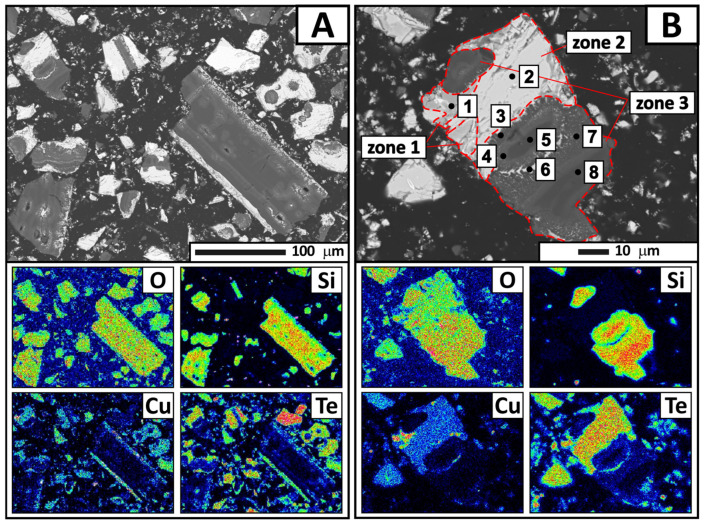
Backscattered electron images of the condensate from the first zone and the distribution of main elements according to electron microanalysis. Magnification: ×250 (**A**) and ×850 (**B**).

**Figure 10:**
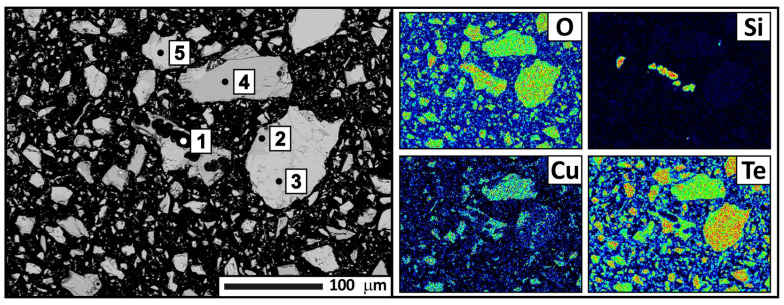
Backscattered electron images of the condensate from the second zone and distribution of the main elements, according to electron microanalysis. Magnification: ×250.

**Figure 11:**
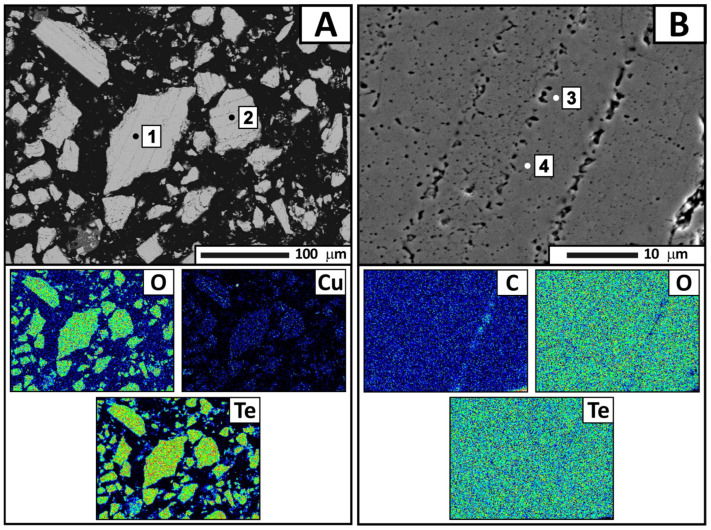
Backscattered electron images of the condensate from the third zone and distribution of the main elements, according to electron microanalysis. Magnification: ×250 (**A**) and ×2000 (**B**).

**Figure 12:**
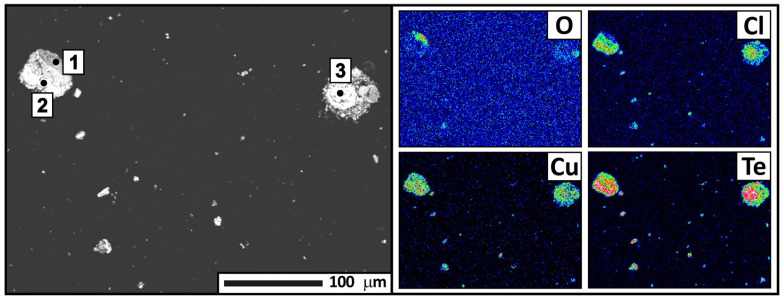
Backscattered electron image of the condensate from the fourth zone and distribution of the main elements, according to electron microanalysis. Magnification: ×250.

For a more detailed analysis, condensate samples were collected from four zones separated by color. The samples were subjected to X-ray phase analysis (Table 3, Figure 8), X-ray fluorescence analysis (Table 4), and electron probe microanalysis (Figure 9, Figure 10, Figure 11 and Figure 12, Table 5, Table 6, Table 7 and Table 8). During interpretation of the point EDS analysis results, the presence of carbon was disregarded, as it is related to sample preparation.

**Table 3:** Phase composition of the condensates by condensation zone.

Zone	Deposition Temperature, °C	CuTe_2_O_5_	TeO_2_	CuO·CuTeO_3_	SiO_2_	CuTe_2_Cl	Te
1	400–350	54.8	19.5	16.6	9.1	–	–
2	350–270	57.7	42.3	–	–	–	–
3	270–150	–	100	–	–	–	–
4	150–100	–	–	–	–	85.5	14.5

As can be seen from the presented data, the condensate from the first zone consists of phases of tellurium-containing compounds (CuTe_2_O_5_, TeO_2_, and CuO·CuTeO_3_) and silica. According to [35], evaporation of silicon oxide is possible only at temperatures above 1700 °C. Therefore, its presence in the condensate as a result of evaporation can be excluded. Its presence in the sample is instead attributed to the collection of condensate from the surface of the quartz condenser. Direct evaporation of CuTe_2_O_5_ and CuO·CuTeO_3_ at the experimental temperature is also unlikely, as these compounds are thermally unstable and decompose with the release of volatile TeO_2_ at lower temperatures. Thus, the presence of tellurites in the condensate can be explained by the intensive evaporation of tellurium dioxide, which mechanically entrains copper oxide molecules. Their deposition occurs together with tellurium oxide in the cold part of the reactor, where secondary formation of the discussed phases is possible.

The electron probe microanalysis results revealed individual condensate grains with heterogeneous microstructure (Figure 9). As shown in the figure, a grain consists of three main zones: light gray (zone 2) and gray (zone 1) zones with a cracked structure, as well as a dark gray zone (zone 3) exhibiting a needle-like structure at the interface with the gray zone.

Point EDS analysis was performed to determine the chemical composition of these specific areas in detail. The results (Table 5) showed that the gray zone corresponds to copper oxide (point 1). The light gray zone contains a significant amount of tellurium (65.02 wt.%), along with copper and oxygen (point 2). This composition indicates the presence of a complex system. The empirical formula calculation yielded a compound of CuTe_2.25_O_5.62_, which is close to CuTe_2_O_5_. The dark gray region consists of oxygen, silicon, tellurium, and copper in varying proportions (points 3–5 and 7–8). The tellurium content ranges from 0.66 to 10.99 wt.%, decreasing as the analysis points move away from the boundaries of the region. It appears that gaseous tellurium-containing compounds interact with amorphous quartz (quartz glass) at condensation temperatures of 350–400 °C. This process is likely accompanied by surface migration of tellurium oxide into the condenser material. Cracking and delamination of the quartz surface layer occur during subsequent crystallization of CuTe_2_O_5_. These processes are apparently responsible for the quartz impurity in the tellurium-containing condensate.

According to the X-ray phase analysis results, the second condensation zone consists of CuTe_2_O_5_ and TeO_2_ phases, with tellurium oxide being the dominant phase. The X-ray fluorescence analysis revealed a significant increase in tellurium content (up to 65.72 wt.%) and a decrease in copper and quartz (down to 5.54 and 0.36 wt.%, respectively). The electron probe analysis results confirmed the X-ray phase analysis data and provided additional information on the presence of quartz (Figure 10, Table 6). CuTe_2_O_5_ occurs both as small inclusions at the boundaries of tellurium oxide grains and as larger isolated formations.

The third zone is represented by a single phase of tellurium oxide with a small amount of copper impurity. The copper content decreases to 0.58 wt.%, while the oxygen and tellurium contents remain almost unchanged (27.058 and 67.202 wt.%, respectively). The sample is relatively homogeneous, with pores and voids of various shapes and sizes observed in the structure (Figure 11, Table 7). Small, round pores are fairly evenly distributed, while larger elongated pores are grouped in straight lines. This defect is associated with layer-by-layer condensation of tellurium oxide during sample preparation and likely resulted from shrinkage of the condensate film upon cooling.

The condensate from the fourth zone is a fine powder, easily detached from the condenser surface. The X-ray phase analysis data indicate that the material mainly contains the CuTe_2_Cl phase with a small amount of tellurium. However, elemental tellurium was not detected in subsequent SEM analysis, likely due to its low content. The main elements forming the condensate are tellurium (35.16 wt.%), copper (23.03 wt.%), oxygen (9.46 wt.%), and chlorine (7.49 wt.%). Electron probe analysis of individual condensate fragments (Figure 12, Table 8) showed that, in addition to the main elements, trace elements from the original sample are also deposited in this zone. The presence of copper in the condensate is most likely associated with the intensive evaporation of volatile tellurium tetrachloride.

Based on the analysis, the following mechanism for the formation of tellurium-containing condensate can be hypothesized. Tellurium chloride and elemental tellurium are initially rapidly evaporated from tellurium-containing middlings. Copper or its chloride compounds are then drawn into the vapor-gas phase, and their combined condensation at low temperatures (150–100 °C) leads to formation of the chlorine-containing CuTe_2_Cl phase. As phase transformations occur in the tellurium-containing middlings, tellurium dioxide is released, and its rapid evaporation is accompanied by the capture of copper oxide molecules. As the vapor-gas phase passes through the first (400–350 °C) and second (350–270 °C) temperature condensation zones, they co-precipitate to form CuTe_2_O_5_ and CuO CuTeO_3_ in the solid phase: Te_2_O + CuO = CuTe_2_O_5_ and Te_2_O + CuO = CuO CuTeO_3_. In the third zone (270-150 °C), residual traces of copper are detected, and the condensate is represented by a single phase of TeO_2_, indicating complete precipitation of copper oxide in the higher-temperature condensation zones.

## 4. Conclusions

The comprehensive set of studies detailed in this paper has, for the first time, presented the tellurium-containing condensate obtained through vacuum–thermal processing of a tellurium-bearing middling in an inert atmosphere. This work also presents new information on the second product of vacuum–thermal treatment (copper-containing residues), obtained under isothermal conditions at low pressure.

It was shown that complete decomposition of copper hydroxysulfates occurs during the process, which provides free oxygen for the oxidation of copper telluride to copper tellurates and tellurites. The residues obtained at 1100 °C and a pressure of 66 Pa are mainly represented by the Cu_2_O phase. The presence of the CuAlO_2_ phase is an impurity. The obtained residues can be subjected to further processing in order to recover metallic copper.

Analysis of the condensate obtained at 1100 °C and 66 Pa revealed the presence of CuTe_2_O_5_, CuO·CuTeO_3_, TeO_2_, SiO_2_, and CuTe_2_Cl. The presence of copper in the condensate is attributed to its mechanical entrainment by volatile tellurium-containing compounds and their co-condensation. In turn, quartz is an impurity introduced into the material during sample collection from the surface of the quartz condenser.

It was shown that, in the 400–300 °C temperature zone, CuTe_2_O_5_, CuO·CuTeO_3_, and TeO_2_ are deposited. In the 350–270 °C zone, CuTe_2_O_5_ and TeO_2_ are deposited. A monophase of tellurium oxide is present in the condensate deposited in the 270–150 °C temperature zone. In the condensate collected from the 150–100 °C zone, the presence of CuTe_2_Cl and elemental tellurium was established, as well as trace elements originating from the initial material.

Contamination of the processing products with impurities from the crucible and condenser is possible due to the formation of a protective (skull) layer on the equipment walls and the replacement of the feedstock container and condenser materials with more stable ones. It is equally important to determine optimal conditions to reduce interaction between the processing products and the plant’s structural components, as well as preventing the removal of copper and its oxide by tellurium-containing vapors.

Thus, the obtained data confirm the feasibility of vacuum–thermal processing tellurium-containing middling in an inert atmosphere without the use of additional reagents. Under these conditions, tellurium is transferred into the condensate predominantly in the form of oxide.

The copper-containing residues can subsequently be processed by well-established methods to extract metallic copper, such as the reduction of carbon-containing reducing agents at elevated temperatures. This approach enables the effective conversion of copper to a metallic state and ensures its return to the production cycle.

To determine the processing direction for tellurium-containing condensate, additional research is needed to determine copper loss by tellurium-containing vapors. In this case, condensate processing is not technologically feasible. The condensate, represented by the TeO_2_ phase, can be subjected to carbothermic reduction at temperatures of 600–700 °C in a vacuum, with the recovery of elemental tellurium. From the condensate, represented by tellurium chlorides, tellurium can be recovered by direct thermal decomposition at temperatures of 300–400 °C in an inert atmosphere.

The obtained data clarify our understanding of vacuum–thermal processing of tellurium-containing products and provide a scientific basis for the development of a new pyrometallurgical method for its processing.

Optimization of the process parameters based on the conducted research will make it possible to justify and propose a new, environmentally safe method for extracting tellurium from tellurium-containing middling derived from copper production.

## Figures and Tables

**Table 1:** Composition of the tellurium-containing middling.

Elemental Composition, wt.%									
Te	Cu	O	S	Cl	Si	As	Al	Se	Pb
23.42	42.45	31.38	2.31	0.20	0.05	0.12	0.02	0.03	0.02

**Table 2:** Elemental composition of residues.

Pressure, Pa	Elemental Composition, wt.%									
	Te	Cu	O	S	Cl	Al	As	Si	Se	Other
133	1.62	48.36	28.39	0.01	0.02	6.04	0.09	12.89	–	2.58
66	0.72	53.13	28.82	0.01	0.01	4.81	0.07	10.31	–	2.13

**Table 4:** Elemental composition of the condensates by condensation zone.

Zone	Elemental Composition, wt.%															
	Te	Cu	O	Si	S	Cl	K	Na	As	Sn	Al	Fe	Pb	Nb	Ti	Se
1	37.70	18.59	29.56	8.06	0.01	0.12	0.14	0.30	0.62	0.27	0.02	–	–	–	–	–
2	65.72	5.54	27.51	0.36	0.07	0.04	0.10	0.19	0.11	0.34	0.01	–	0.03	0.01	–	–
3	67.20	0.58	27.06	0.20	0.14	–	0.15	0.11	0.03	0.45	0.02	–	0.03	–	–	0.01
4	35.16	23.03	9.46	0.23	1.04	7.49	–	–	0.14	–	0.02	0.05	–	0.01	0.13	2.05

**Table 5:** Point EDS analysis of the condensate from the first zone.

Element	Content at the Analysis Point, wt.%							
	1	2	3	4	5	6	7	8
Te	–	65.02	7.24	10.99	0.66	50.04	4.84	1.82
Cu	85.83	14.47	2.80	3.12	–	11.75	–	–
O	14.07	20.51	53.33	51.48	55.95	27.77	55.69	55.27
Si	–	–	36.63	34.41	43.39	7.08	39.47	42.91
C	–	–	–	–	–	3.36	–	–

**Table 6:** Point EDS analysis of the condensate from the second zone.

Element	Content at the Analysis Point, wt.%				
	1	2	3	4	5
Te	–	62.22	76.27	61.99	76.15
Cu	–	13.17	–	13.51	–
O	59.56	22.00	21.67	21.93	21.53
Si	40.44	–	–	–	–
C	–	2.61	2.06	2.57	2.32

**Table 7:** Point EDS analysis of the condensate from the third zone.

Element	Content at the Analysis Point, wt.%			
	1	2	3	4
Te	81.08	80.63	80.19	77.39
Cu	–	0.14	–	–
O	18.92	19.23	19.81	19.89
C	–	–	–	2.71

**Table 8:** Point EDS analysis of the condensate from the fourth zone.

Element	Content at the Analysis Point, wt.%		
	1	2	3
Te	54.05	70.03	72.35
Cu	16.66	18.92	16.22
O	20.36	–	1.01
Al	0.63	–	–
Si	0.60	–	–
S	1.75	–	–
Cl	2.02	9.41	8.73
K	0.02	–	–
Cr	3.31	–	0.68
Se	0.95	1.64	1.01

## Data Availability

The original contributions presented in this study are included in the article. Further inquiries can be directed to the corresponding author.
